# Draft Genome Sequences of *Chryseobacterium artocarpi* UTM-3^T^ and *Chryseobacterium contaminans* C26^T^, Isolated from Rhizospheres, and *Chryseobacterium arthrosphaerae* CC-VM-7^T^, Isolated from the Feces of a Pill Millipede

**DOI:** 10.1128/genomeA.01168-16

**Published:** 2016-10-20

**Authors:** Jin-Ju Jeong, Byeonghyeok Park, Ji Yeon Oh, Mohamed Mannaa, Yoo Jun Kim, Jeum Kyu Hong, In-Geol Choi, Ki Deok Kim

**Affiliations:** a Laboratory of Plant Disease and Biocontrol, College of Life Sciences and Biotechnology, Korea University, Seoul, Republic of Korea; b Department of Biotechnology, Korea University, Seoul, Republic of Korea; c Department of Horticultural Science, Gyeongnam National University of Science and Technology, Jinju, Republic of Korea

## Abstract

Species of the genus *Chryseobacterium* belonging to the family *Flavobacteriaceae* are nonmotile, yellow-pigmented, and rod-shaped bacteria, some of which were frequently isolated from soil or plant-related materials. Here, we present draft genome sequences of three type strains of *Chryseobacterium*, which contain genes related to plant growth promotion, colonization, or stress adaptation.

## GENOME ANNOUNCEMENT

To date, 95 species of *Chryseobacterium* have been identified, and these are abundant in diverse environments, such as rhizospheres or soil, water, plant materials, and chicken **(**1–8). Among *Chryseobacterium* species, some have been shown to exhibit biocontrol activity against plant pathogens as well as plant growth promotion activity (3, 7–9). Previously, *Chryseobacterium* sp. strain GSE06 (tentatively identified as *Chryseobacterium gunsanense* strain GSE06) was proven to exhibit biocontrol activity against a destructive soilborne oomycete pathogen, *Phytophthora capsici*, on pepper (8, 10). In a phylogenetic analysis for molecular identification, strain GSE06 was grouped with *Chryseobacterium arthrosphaerae* CC-VM-7^T^, isolated from the feces of the pill millipede (*Arthrosphaera magna*) (11); *Chryseobacterium artocarpi* UTM-3^T^, isolated from the rhizosphere of *Artocarpus integer* (6); and *Chryseobacterium contaminans* C26^T^, isolated from a rhizosphere sample (5). These species may also have similar activities, such as plant growth promotion, colonization, and stress management, as observed in strain GSE06 (8). Here, we report the draft genome sequences of *C. arthrosphaerae* CC-VM-7^T^, *C. artocarpi* UTM-3^T^, and *C. contaminans* C26^T^.

Genomes of strains CC-VM-7^T^, UTM-3^T^, and C26^T^ were sequenced using the Illumina MiSeq platform at the Computational and Synthetic Biology Laboratory, Korea University (Seoul, South Korea). A total of 1,440,261, 1,622,828, and 2,310,401 paired-end reads (864.2, 973.7, and 1,386.2 Mb, and 164.9, 186.4, and 281.2-fold coverage) for strains CC-VM-7^T^, UTM-3^T^, and C26^T^, respectively, were generated from paired-end sequencing of the genomic library, with an average insert size of 500 bp. Low-quality reads were trimmed, using a quality threshold of Q30, and then subjected to *de novo* assembly, using the SPAdes assembler (12). The reads were assembled to 36, 51, and 366 scaffolds for strains CC-VM-7^T^, UTM-3^T^, and C26^T^, respectively, with total lengths and G+C contents as shown in Table 1. The *N*_50_ values of the contigs were 3,426,081, 718,111, and 25,005 bp for strains CC-VM-7^T^, UTM-3^T^, and C26^T^, respectively. Genome annotation was performed using the NCBI Prokaryotic Genome Annotation Pipeline (PGAP) service. The total predicted coding sequences and the retrieved numbers of tRNA, 5S rRNA, 16S rRNA, and 23S rRNA sequences of the strains are shown in Table 1. 

**TABLE 1:** Summary of genome sequencing and GenBank accession numbers of the type strains of three *Chryseobacterium* species

Strain	Genome size (bp)	G+C content (%)	No. of coding sequences	No. of tRNAs	No. of rRNAs			Accession no.
					5S	16S	23S	
*C. arthrosphaerae* CC-VM-7^T^	5,053,343	38.3	4,445	77	1	4	5	MAYG00000000
*C. artocarpi* UTM-3^T^	4,935,607	34.8	4,307	77	6	1	1	MAYH00000000
*C. contaminans* C26^T^	4,704,265	35.9	4,000	79	4	3	1	MAYF00000000

The genomes of strains CC-VM-7^T^, UTM-3^T^, and C26^T^ contain genes related to plant growth promotion, such as those encoding siderophores, urease, nitrogen fixation proteins, and 1-aminocyclopropane-1-carboxylate (ACC) deaminase (13–15). Furthermore, strains UTM-3^T^ and C26^T^ have colonization genes, such as the gene encoding diguanylate cyclase, and strain CC-VM-7^T^ has environmental stress management genes, such as those encoding superoxide dismutase and hydrogen peroxidase (16). In conclusion, genome analyses of *C. arthrosphaerae* CC-VM-7^T^, *C. artocarpi* UTM-3^T^, and *C. contaminans* C26^T^ will be helpful to understand the biological traits of the strains, such as plant growth promotion, colonization, and environmental stress adaptation.

### Accession number(s).

This whole-genome shotgun project has been deposited in the DDBJ/EMBL/GenBank under the accession numbers listed in Table 1. The versions described in this paper are the first versions.
